# Crystallization of Poly(ethylene)s with Regular Phosphoester Defects Studied at the Air–Water Interface

**DOI:** 10.3390/polym12102408

**Published:** 2020-10-19

**Authors:** Nazmul Hasan, Karsten Busse, Tobias Haider, Frederik R. Wurm, Jörg Kressler

**Affiliations:** 1 Institute of Chemistry, Martin Luther University Halle-Wittenberg, D-06099 Halle, Germany; nazmul.hasan@chemie.uni-halle.de (N.H.); karsten.busse@chemie.uni-halle.de (K.B.); 2 Max Planck Institute for Polymer Research, Ackermannweg 10, D-55128 Mainz, Germany; haider@mpip-mainz.mpg.de; 3 Sustainable Polymer Chemistry Group, MESA+ Institute for Nanotechnology, Faculty of Science and Technology, Universiteit Twente, P.O. Box 217, 7500 AE Enschede, The Netherlands; frederik.wurm@utwente.nl

**Keywords:** poly(ethylene), Langmuir–Blodgett film, crystallization, AFM and GI-WAXS

## Abstract

Poly(ethylene) (PE) is a commonly used semi-crystalline polymer which, due to the lack of polar groups in the repeating unit, is not able to form Langmuir or Langmuir–Blodgett (LB) films. This problem can be solved using PEs with hydrophilic groups arranged at regular distances within the polymer backbone. With acyclic diene metathesis (ADMET) polymerization, a tool for precise addition of polar groups after a certain interval of methylene sequence is available. In this study, we demonstrate the formation of Langmuir/LB films from two different PEs with regular phosphoester groups, acting as crystallization defects in the main chain. After spreading the polymers from chloroform solution on the water surface of a Langmuir trough and solvent evaporation, the surface pressure is recorded during compression under isothermal condition. These *π*-*A* isotherms, surface pressure *π* vs. mean area per repeat unit *A*, show a plateau zone at surface pressures of *~* (6 to 8) mN/m, attributed to the formation of crystalline domains of the PEs as confirmed by Brewster angle and epifluorescence microscopy. PE with ethoxy phosphoester defects (Ethoxy-PPE) forms circular shape domains, whereas Methyl-PPE-*co*-decadiene with methyl phosphoester defects and two different methylene sequences between the defects exhibits a film-like morphology. The domains/films are examined by atomic force microscopy after transferring them to a solid support. The thickness of the domains/films is found in the range from *~* (2.4 to 3.2) nm depending on the transfer pressure. A necessity of chain tilt in the crystalline domains is also confirmed. Grazing incidence X-ray scattering measurements in LB films show a single Bragg reflection at a scattering vector q_xy_ position of *~* 15.1 nm^−1^ known from crystalline PE samples.

## 1. Introduction

**Scheme 1:**
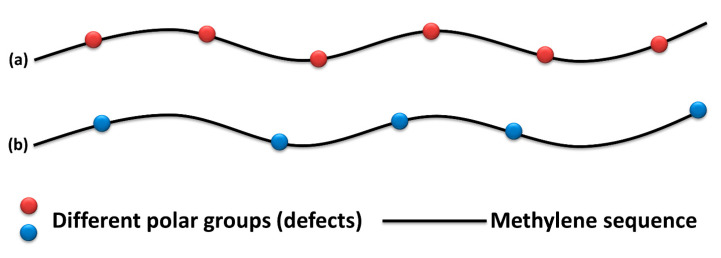
Schematic illustration of (**a**) Ethoxy-PPE and (**b**) Methyl-PPE-*co*-decadiene.

Polymer crystallization in thin films has been studied with respect to basic research [1,2,3,4,5,6,7,8] and applications [9,10] for the last few decades. Various polymer properties change with film thickness compared to their bulk state influencing polymer crystallization as molecular mobility [11,12], glass transition temperature [3,13,14,15,16,17,18], and chain orientation [19,20]. Thin solid films are usually prepared by spin coating a polymer solution onto a solid substrate [11,21]. An alternative approach for the preparation of polymer thin films is spreading the polymer solution on aqueous surfaces of a Langmuir trough, followed by solvent evaporation [22,23]. These films are also known as Langmuir films [24]. Finally, Langmuir film compression yields various film thicknesses in the Å- to nm-range connected with different states of order. This method also provides the option to control crystallization kinetics by changing parameters of the Langmuir trough as the barrier compression–expansion speed and subphase temperature or even replacing the water subphase with aqueous salt solutions [25]. The formation of butterfly-like crystals of poly(ε-caprolactone) (PCL) [26] or dendritic morphology of poly(ethylene oxide) (PEO) [25] Langmuir films are notable. The Langmuir films can be transferred to solid supports using the Langmuir–Blodgett (LB) technique [24]. The LB films allow to study the surface morphology with high lateral resolution, e.g., by atomic force microscopy (AFM). The observation of the double helix of isotactic poly(methyl methacrylate) (i-PMMA) is one of the most impressive examples [27,28]. A major drawback for the preparation of Langmuir films and finally LB films is the limited number of suitable polymers [22,23]. They must contain a polar group in the repeating unit, which helps to anchor the polymer chain to the water surface to avoid immediate collapse [29]. Some polymers such as PCL, poly(L-lactic acid) (PLA), and i-PMMA fulfill this requirement with their ester groups and are used for the preparation of Langmuir or LB films [26,27,30,31]. However, Langmuir or LB film formation of poly(ethylene) (PE) was not successful and crystallization studies were not reported. Some thin film studies have been done using medium-density PE [32,33]. The issue regarding PE Langmuir/LB film formation is the lack of polar groups in the repeating unit. Different approaches are reported to introduce polar groups to PE such as surface modification of PE [34,35], copolymerization [36], and living polymerization [37]. Some approaches yield highly branched PEs with randomly distributed polar groups in the polymer backbone or side chain with high polydispersities [38]. Acyclic diene metathesis (ADMET) polymerization [39] allows to synthesize linear PE-like materials with polar groups (defects) in the polymer backbone (Scheme 1) with moderate polydispersities [40,41]. These polymers are called precision polymers [40,41]. Their crystallization behavior in bulk and solvent cast films or melt grown domains has already been reported in detail elsewhere [41,42]. The crystallization of such a polymer on the surface of water has been reported by our group. We reported the crystallization of a PE with a phosphoester defect in the main chain at every 21st position of the CH_2_ backbone (Phenoxy-PPE) at the air-water interface [43]. After spreading this polymer from the chloroform solution on the surface of water, some polymer chains immediately crystallize at *π* of 0 mN/m, but the final crystallization of most of the polymer chains occurs upon compression in the expanded plateau zone of the Langmuir isotherm (*π ~* 4.5 mN/m). Crystallization is monitored directly on the surface of water with Brewster angle microscopy (BAM) and epifluorescence microscopy. Single crystallites with hexagonal shape are observed. Most of the crystallites have a thickness of *~* 2.6 nm with an elevated region in the center of the crystal measured in LB films [43].

In this study, Langmuir films of different PEs namely Ethoxy-PPE and Methyl-PPE-*co*-decadiene (see Table 1) are prepared by spreading the polymer solutions on the water surface of a Langmuir trough and compressing them after solvent evaporation. The morphology of the films is monitored by BAM and epifluorescence microscopy during the compression. Finally, LB films are prepared by transferring the film from the water surface to a solid support to study the film thickness by atomic force microscopy (AFM) and crystallization by grazing incidence wide-angle X-ray scattering (GI-WAXS).

## 2. Materials and Methods

### 2.1. Materials

PEs with regular phosphoester defects in the main chain under investigation are listed in Table 1. All polymers were synthesized by ADMET polymerization. Ethoxy-PPE has 20 CH_2_ units in each repeat unit. Methyl-PPE-*co*-decadiene contains randomly (20 to 28) CH_2_ units in every repeat unit since it is a (1:1) random copolymer obtained by copolymerization of an equimolar mixture of 1,9-decadiene and di(undec-10-en-1-yl) methylphosphonate. The synthesis of the Ethoxy-PPE is described elsewhere [42]. Characterization data for the Methyl-PPE-*co*-decadiene copolymer can be found in the supporting information (Figures S1–S3). All polymers are semi-crystalline in bulk and thin films, where only the methylene chains crystallize [41,42]. The DSC traces, the X-ray diffraction (XRD) patterns, and the FTIR spectra of the polymers can be found in Figures S4–S5 of the supporting information.

### 2.2. Langmuir Isotherms Measurement

The *π*-*A* isotherms were recorded using a Langmuir trough (Riegler & Kirstein GmbH, Potsdam, Germany) with a maximum trough area of 545 cm^2^. The trough was equipped with two moveable barriers and a Wilhelmy plate made of filter paper. The entire trough was covered by a Plexiglas box to maintain an equilibrium environment. Millipore water was used as a subphase for the experiment. The temperature of the subphase was kept at 20 °C using a thermostat. Before spreading the polymer solution, the purity of the subphase was checked by surface pressure measurement at maximum barrier compression (*π* < 0.15 mN/m). Polymer solutions with a concentration of ~2 mg/mL were prepared in chloroform and spread dropwise in some random locations on the subphase using a Hamilton digital syringe. After a 20 min waiting time for complete solvent evaporation, the trough surface was compressed at a speed of 50 Å^2^/(molecule min) to record the pressure–area isotherm.

### 2.3. Microscopic Studies on Langmuir Films

To monitor the water surface during compression, a Brewster angle microscope (NFT Mini BAM, Nanofilm Technologies, Valley View, OH, USA) coupled with a Langmuir trough of 142 cm^2^ was used. The lateral resolution of the microscopy was 20 μm with a field view of 4.8 × 6.4 mm^2^. The images were captured using the software WinTV (Hauppauge Inc, Hauppauge, NY, USA). The imaging of the Langmuir film was done at different surface pressures during the film compression at a rate of 50 Å^2^/(molecule min). Epifluorescence images were recorded with an Axio Scope A1 Vario epifluorescence microscope (Carl Zeiss MicroImaging, Jena, Germany). The microscope was equipped with an EC Epiplan-NEOFLUAR 50x objective and a Hamamatsu EM-CCD digital camera. A film balance (Riegler & Kirstein GmbH, Potsdam, Germany) with a maximum trough area of 258 cm^2^ covered with a Plexiglas chamber was used. The temperature of the subphase was kept at 20 °C. To enable the experiment, the aqueous subphase contained 50 nM Rhodamine B fluorescence dye ([9-(2-carboxyphenyl)-6-diethylamino-3-xanthenylidene]-diethylammonium chloride). The dye was excited using a 100 W mercury arc lamp through a combination of BP 546/12 nm window and a beam splitter FT 560 nm. The emission was detected via a BP 575–640 nm (filterset 20, Carl Zeiss AG, Jena, Germany). The imaging of the monolayer was done during the film compression with a speed of 50 Å^2^/(molecule min) at different surface pressures.

### 2.4. Langmuir–Blodgett (LB) Film Transfer, AFM, and GI-WAXS

**Figure 1:**
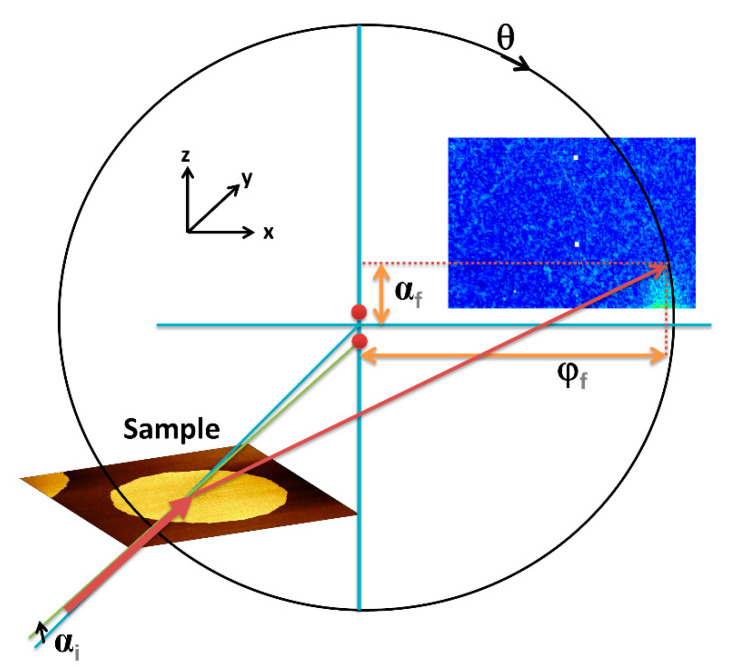
Sample geometry for GI-WAXS measurements.

Silicon wafer with a size of 20 × 10 mm^2^ was cleaned and mounted to a film transfer unit (KSV Instruments, Helsinki, Finland) to prepare LB films. The film was made at *π* of 10 mN/m and 15 mN/m, respectively. The substrate was attached vertically to the transfer unit and immersed into the subphase ~8 mm. The polymer solution was then spread on the water surface and compressed up to the transfer pressure. When the transfer pressure was reached, the submerged silicon substrate was moved upward at a speed of 0.5 mm/min, while the surface pressure was kept constant. This process transfers a film of the polymer on the substrate surface. The film was then dried at room temperature and stored in a sealed box for AFM and GI-WAXS measurements. AFM studies were carried out in AC mode by NanoWizard 4 (JPK, Berlin, Germany) instrument in air. A silicon cantilever with a spring constant of 40 N/m and a resonance frequency of 325 kHz was used. The captured images were then processed by JPK and Gwyddion software. A Retro-F SAXSLAB setup (SAXSLAB, Copenhagen, Denmark) equipped with an AXO microfocus X-ray source (AXO DRESDEN GmbH, Dresden, Germany) and a DECTRIS PILATUS3 R 300K detector (DECTRIS Ltd, Baden-Daettwil, Switzerland) was used to perform GI-WAXS measurements on the LB films of PEs. Figure 1 provides a schematic presentation of the setup used. Measurements were conducted at room temperature under vacuum condition in reflection mode. The incidence angle *α_i_* of CuKα radiation (λ = 1.5418 Å) was ~0.2°. The detector images were converted to sample coordinates according to Equation (1) and the condition $n_{XR}=\cos\left(\alpha_{c}\right)\cos\left(\alpha_{i}\right)1\;$ with the critical angle *α_c_* [26].

(1) $$\left(\begin{array}{c} q_{x}\\ q_{y}\\ q_{z} \end{array}\right)=\frac{k_{0}}{n_{XR}}\left(\begin{array}{c} \cos\varphi_{f}\cos\alpha_{f}-n_{XR}\\ \sin\varphi_{f}\cos\alpha_{f}\\ \sqrt{n_{XR}2-\cos^{2}\alpha_{f}} \end{array}\right)$$

where *q_x_*, *q_y_*, and *q_z_* are the scattering vector in *x*, *y*, and *z* coordinates, $n_{XR}$ is the refractive index, $k_{0}$ is the wavenumber, $\alpha_{f}$ and $\varphi_{f}$ are the vertical and horizontal scattering angle, respectively.

The horizontal part of the scattering vector q is given with $q_{xy}=\sqrt{q_{x}^{2}+q_{y}^{2}}$.

## 3. Results and Discussion

**Figure 2:**
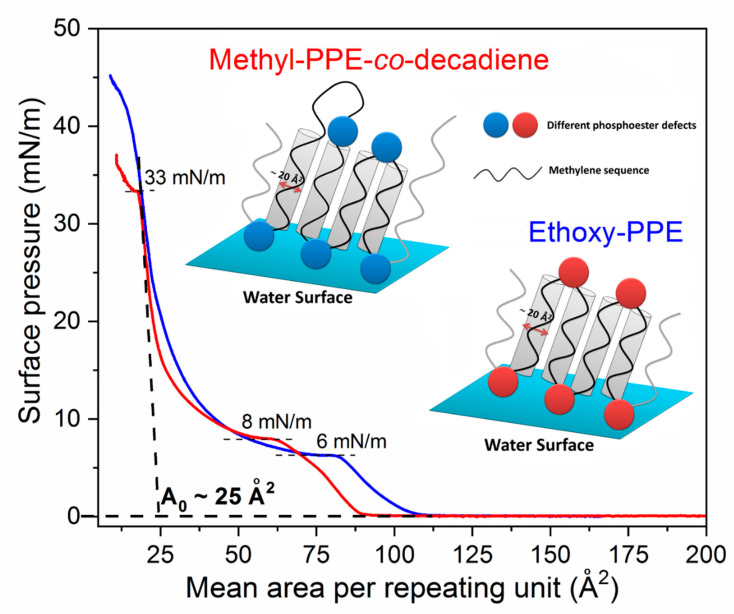
*π*-*A* isotherms of two different PEs measured at a subphase temperature of 20 °C with a compression rate of 50 Å^2^/(molecule min). The insets show the possible chain orientation of the PEs at *A_0_* position.

Figure 2 depicts the surface pressure vs. mean area per repeat unit (*π*-*A*) isotherms of two different PEs that are recorded after spreading the polymers from chloroform solution on the water subphase of a Langmuir trough to *π =* 0 mN/m (*A* ~ 200 Å^2^) and compressing them with a rate of 50 Å^2^/(molecule min) after solvent evaporation under isothermal condition.

The isotherm of Ethoxy-PPE shows that with decreasing *A*, the *π* begins to increase from an *A* value of ~106 Å^2^, followed by an extended plateau region at ~6 mN/m and finally reaches ~45 mN/m. The isotherm is slightly different for Methyl-PPE-*co*-decadiene, with an increase of *π* starting at ~90 Å^2^ and reaches finally a surface pressure of ~37 mN/m. The plateau appears at a slightly higher pressure of ~8 mN/m and a kink at a surface pressure of ~33 mN/m. Thus, both PEs form Langmuir films where both PEs adopt different phases such as an amorphous film phase before the plateau and a crystalline solid phase after the plateau as already known from Phenoxy-PPE [43]. Therefore, the plateaus are obviously related to a 1st order phase transition from an amorphous phase to a crystalline state. Both plateaus end with a limiting area per repeating unit *A*_0_ of ~25 Å^2^. This is calculated by drawing a tangent to the Langmuir isotherm after the crystallization plateau and extrapolation to zero surface pressure. The value is almost equal to the space required for a phosphate group (~24 Å^2^) [44,45], but significantly larger than the area of a methylene sequence in zig-zag conformation (19 Å^2^) or in the rotator phase (21 Å^2^) [46]. Since the CH_2_ sequences are not sufficiently long for back folding, more likely is a Langmuir monolayer formation for PEs where every second phosphoester defect is located on the water surface, while the hydrophobic methylene sequences (20–28 CH_2_ units) avoid water contact in the air. They are aligned slightly tilted to the surface of the water as will be discussed in detail below. Every second phosphoester defect is placed at the polymer–air interface (see the insets of Figure 2). This orientation might be slightly different for Methyl-PPE-*co*-decadiene since the methylene sequences have a different length between the defects (see the insets of Figure 2). Finally, the area ratio of the methylene chain, e.g., at *A*_0_ ~ 25 Å^2^, in zig-zag conformation of 19 Å^2^ or in the rotator phase of 21 Å^2^, gives an average tilt angle of 37° (arccos 19 Å^2^/25 Å^2^ or 21 Å^2^/25 Å^2^) with respect to the surface normal. Note that chain tilt is a very common phenomenon in Langmuir films and has been described extensively for polymers [26] and small molecules [47]. This can also be found in crystalline lamellae of oligomers [48] as well as polymers in bulk [49] and thin-films [50]. Chain tilt is typically caused by chain folding [49], the presence of defects in the main chain [48], or packing of end groups [48]. The chains in the polymer crystals reach a higher density than the amorphous chains due to the chain tilt. This has been confirmed for PE and discussed in detail elsewhere [49]. In the case of Langmuir films, a common reason for chain tilting is the packing of head groups. It is observed that molecules with bulky head groups often form tilted conformations of long alkyl chains in Langmuir films [51,52]. Here, the bulky head groups hinder the chain packing perfectly normal to the surface. Thus, the molecules must be tilted to preserve the close contact between the chains in crystallographic order [51]. Furthermore, chain tilting in Langmuir film depends on the compression state, e.g., PCL under various compressional conditions shows a chain tilt in the rage of 21° to 38° [26]. Altogether, one can say that the observed limiting area values confirm the monolayer formation of these hydrophobic polymers with tilted methylene chains normal to the water surface and the polar chain defects which act as anchor groups to the water surface. In contrast, hydrophobic polymers show an extremely small limiting in the range of (0.2 to 2) Å^2^ [53], related to 3D film formation or aggregation [29,54].

**Figure 3:**
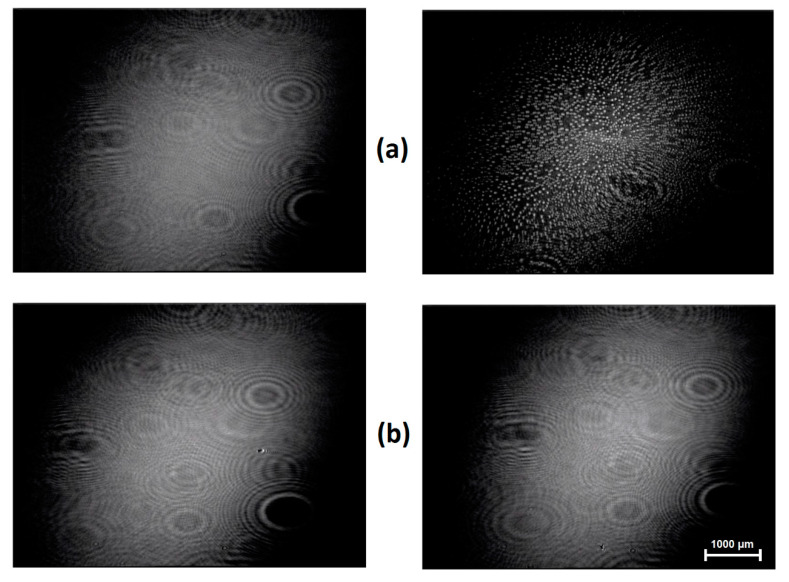
Brewster angle microscopy (BAM) images of (**a**) Ethoxy-PPE at *π* of 2 mN/m, *A* ~ 96 Å^2^ (left) and 7 mN/m, *A* ~ 63 Å^2^ (right), and (**b**) Methyl-PPE-*co*-decadiene at *π* of 2 mN/m, *A* ~ 84 Å^2^ (left) and 9 mN/m, *A* ~ 47 Å^2^ (right). All images were captured with a compression rate of 50 Å^2^/(molecule min). The image size is 4.8 × 6.4 mm^2^.

Now, various microscopic techniques will be used to observe the film morphology of the PEs on the water surface. Figure 3 shows BAM images of the Langmuir film of PEs captured during the compression.

No morphological features are observed before reaching the plateaus of the Langmuir isotherms of both PEs, indicating a homogeneous film formation within the lateral resolution of the BAM equipment (Figure 3a, b, left-side images). When the plateau region is reached during the compression, the Ethoxy-PPE shows many bright domains (Figure 3a, right-side image). These domains are separated from each other, indicating a two-phase system with the solid tiny domains surrounded by the thin liquid film. For Methyl-PPE-*co*-decadiene sample, no domain formation is observed before and after the plateau region is reached (Figure 3b images), even compressing the film up to its collapse phase at *π* of ~33 mN/m. The kink at ~33 mN/m of this polymer is assigned to film collapse by BAM (see Figure S6 of supporting information). Note that domains formation in Langmuir experiments for semi-crystalline polymers is typically related to crystallization [43,54] and can be resolved by BAM, e.g., butterfly-like crystals of PCL [54]. In our case, the domains are too small to identify their exact morphology by BAM (Figure 3a, right-side image). Thus, epifluorescence microscope with the magnification of 40 times compared to BAM is employed for further investigations. A 50 nM aqueous solution of Rhodamine-B dye was used as subphase. Here, initially a bright contrast was observed from the dye subphase, but a dark contrast was generated when the domains appear upon compression. This is due to the exclusion of the dye from domains caused by crystallization [55,56,57,58]. Note that the dye molecules are just like an impurity in the subphase, which might influence the *π*-*A* isotherms or even the domain morphology [55,56,57,58]. In our case, no influence of the dye subphase on the *π*-*A* isotherm was detected (supporting information Figure S7).

**Figure 4:**
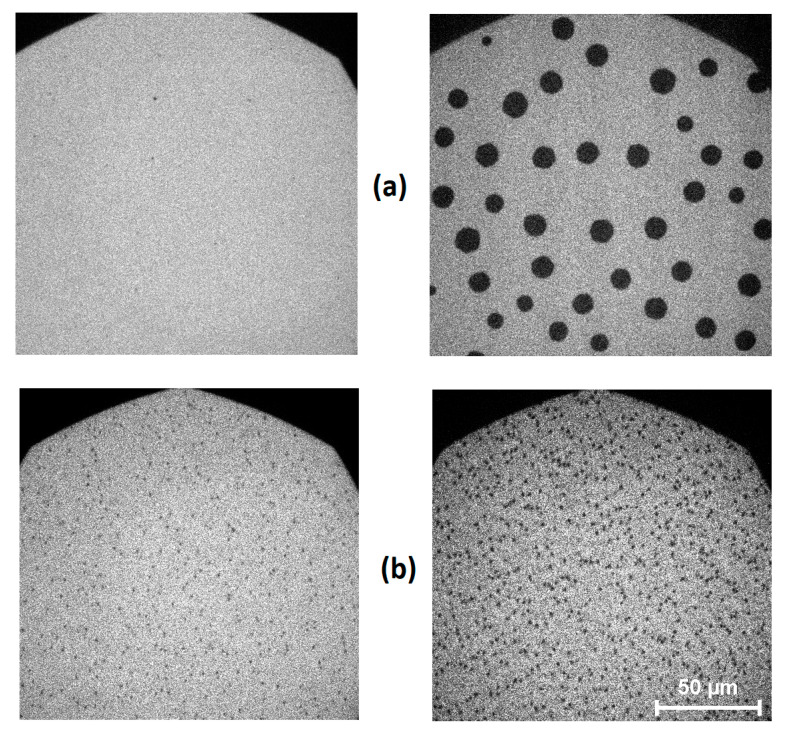
Epifluorescence images of (**a**) Ethoxy-PPE at *π* of 2 mN/m, *A* ~ 96 Å^2^ (left) and 7 mN/m, *A* ~ 63 Å^2^ (right), and (**b**) Methyl-PPE-*co*-decadiene at *π* of 2 mN/m, *A* ~ 84 Å^2^ (left) and 9 mN/m, *A* ~ 47 Å^2^ (right). The images are captured during the film compression at a compression rate of 50 Å^2^/(molecule min). The image size is 164 × 164 µm^2^.

Figure 4 shows epifluorescence images of the PEs recorded on the dye subphase at two different compression states. These images resolve the morphology of the domains formed at the plateau zone and simultaneously indicate some tiny pre-domain formation before reaching the plateau region of the Langmuir isotherms. We observe some dark spots (Figure 4a,b, left-side images) after spreading the polymers solution on the dye subphase, solvent evaporation, and compressing them to initial increase of the surface pressure at 2 mN/m. For the Methyl-PPE-*co*-decadiene sample, the number of spots is larger and more clearly visible compared to Ethoxy-PPE. Note that the dark contrast of the domain is typically related to crystallization caused by the dye exclusion [43,55,56,57,58]. Thus, some crystallization may occur for both PEs immediately after spreading the polymer solutions and solvent evaporation. This behavior has also been observed for arachidic acid (C20 compound) [59]. In our case, we cannot confirm crystallization at this compression state by GI-WAXS experiment since this method is not sensitive enough for these small amounts of crystalline material. Continuing the film compression from 2 mN/m to the beginning of the plateau of the *π*-*A* isotherm, no detectable changes on the subphase surface are observed. When the plateau starts upon compression, lots of dark domains are observed (not shown here). Upon further compression, these domains become large and the morphology becomes distinguishable, e.g., circular- or hexagonal-shaped domains of Ethoxy-PPE appear (Figure 4a, right-side image). Methyl-PPE-*co*-decadiene forms too small domains that are still difficult to resolve by epifluorescence microscopy (Figure 4b, right-side image). Therefore, the domains of both PEs are transferred from the water surface to silicon wafer and examined by AFM.

**Figure 5:**
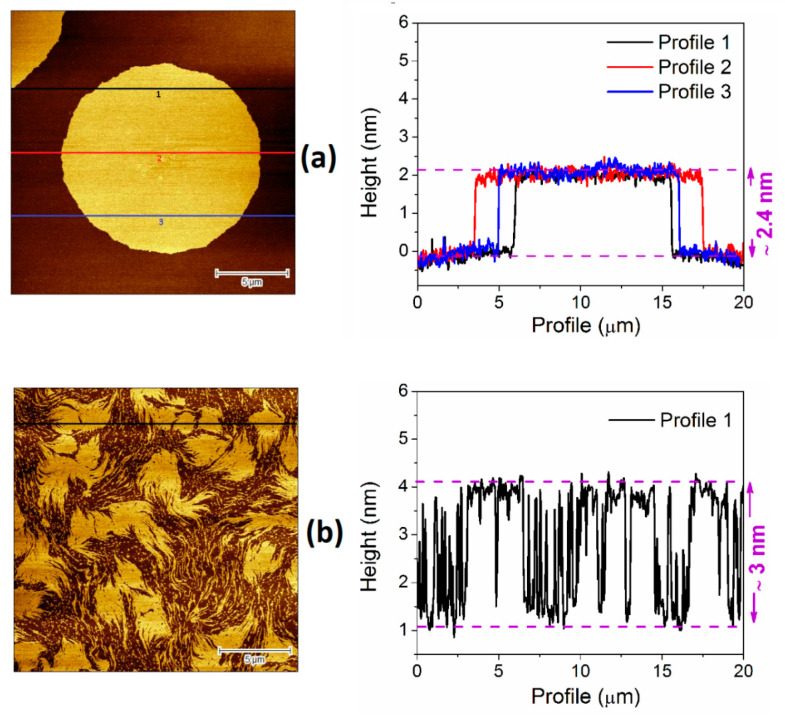
AFM height images (left) with the line profiles (right) of the LB film transferred at a *π* of 10 mN/m for (**a**) Ethoxy-PPE mN/m and (**b**) Methyl-PPE-*co*-decadiene. The image size is 20 × 20 µm^2^. Enlarged AFM images of Methyl-PPE-*co*-decadiene can be found in Figure S8 of the supporting information.

**Figure 6:**
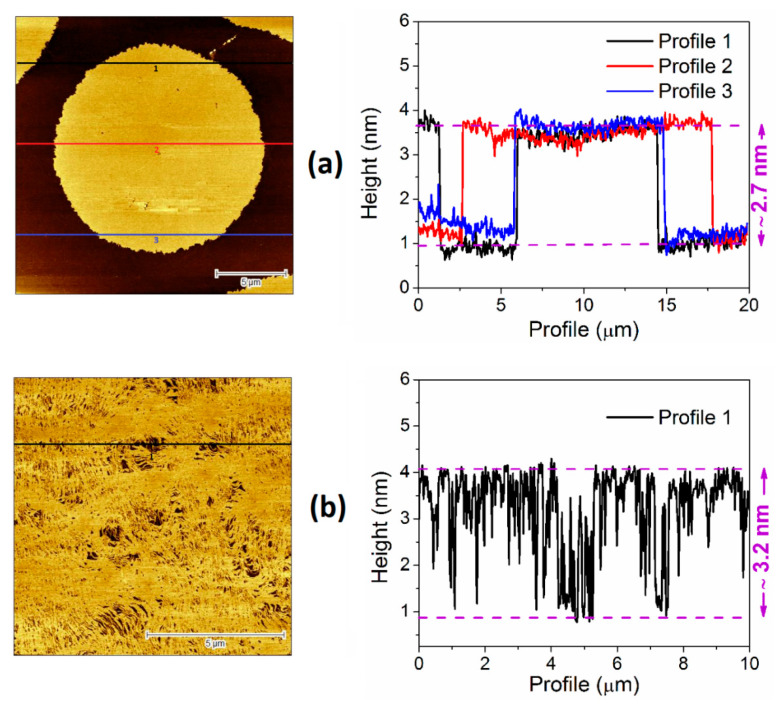
AFM height images (left) with the line profiles (right) of the LB film transferred at a *π* of 15 mN/m for (**a**) Ethoxy-PPE mN/m and (**b**) Methyl-PPE-*co*-decadiene.

Figure 5 shows AFM height images of the PE LB films transferred at 10 mN/m. A circular-shaped crystalline domain with a diameter of more than 15 µm is observed for Ethoxy-PPE (Figure 5a). Note that PE typically forms a lozenge-shaped single crystal due to the slowest growth of {110} planes [60]. There are also a truncated lozenge-shaped or lenticular-shaped crystal habits associated with supercooling temperature-dependent growth rates of the crystal planes [61,62]. Obviously, other factors are also involved in different crystal habits such as geometrical confinement [63], soft epitaxy [64], or molecular fractionation [61,65]. For the Methyl-PPE-*co*-decadiene sample, the morphology is very different compared to Ethoxy-PPE. Completely irregular-shaped large domains surrounded by small domains are observed (Figure 5b). These small domains can be related to the low molar mass fraction of the polymer as known from PE [61,65]. All domains together form a film-like morphology when the LB film is transferred at slightly higher surface pressure (Figure 6b). The irregularity in Methyl-PPE-*co*-decadiene domains can also be explained based on polymer stiffness. The stiffer backbone of Methyl-PPE-*co*-decadiene together with the random distribution of the length of the methylene sequences may hinder the growth of large regular domains. Note that Methyl-PPE-*co*-decadiene is stiffer than the Ethoxy-PPE known from *T_g_* data as measured by DSC (see Figure S4 of the supporting information). Chain length-dependent morphologies of melt/solution grown crystals also show tiny irregular crystallites for Ethoxy-PPE with 20 CH_2_ units [42]. This changes to a dendritic or branch-like feature when the chain length between the Ethoxy-PPE defects increases to 40 CH_2_ units [42]. It can be concluded that the morphologies of the PEs in Langmuir and finally in LB films can be tuned by increasing the length of the methylene sequence in between the phosphoester defects.

Domain/film thicknesses are also estimated by drawing line profiles on the AFM images of the LB films. A maximum value in the range of ~(2.4 to 3) nm is observed (see Figure 5). This value is almost equal to the length of the methylene sequence (20 to 28 CH_2_ units) in a fully extended state. However, the theoretically calculated maximum length for Ethoxy-PPE is ~3.5 nm. This is estimated considering two phosphoester defects (one defect is ~0.5 nm) on both sides of the 20 CH_2_ groups (2.5 nm) with a fully extended state (see discussion below). The small thickness obtained by AFM measurements might have two possibilities; either the phosphoester defects might be accommodated inside the crystalline domains, or the polymer chains in the domains are tilted by some degrees with respect to the substrate normal. Thus, the length ratio, e.g., thickness from AFM (2.4 nm) and the theoretically calculated maximum length (3.5 nm), gives a chain tilt of ~47^o^ (arccos 2.4 nm/3.5 nm) with respect to the substrate normal. The value is also consistent with the chain tilt angle value calculated from the limiting area of the *π*-*A* isotherm. The chain tilt can be further analyzed when the domains/films are transferred at a slightly higher surface pressure of ~15 mN/m (see Figure 6a). A slight increase (2.7 nm − 2.4 nm = 0.3 nm) in domain thickness is observed which might be related to the decrease in the chain tilt from ~47° to ~40° (arccos 2.7 nm/3.5 nm, i.e., thickness from AFM (2.7 nm) divided by the theoretically calculated maximum length (3.5 nm)) due to the compression.

**Figure 7:**
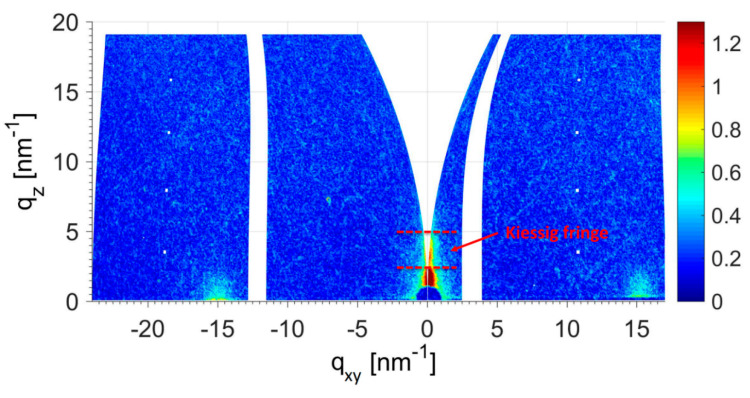
2D GI-WAXS pattern of the LB film of Ethoxy-PPE transferred at 15 mN/m (after the plateau zone) of the *π*-*A* isotherm. The two vertical bands in the image are the empty space between the detectors and appear as blank. The dotted red lines are drawn in to the q_z_ axis to guide the range of the Kiessig fringe.

Finally, GI-WAXS experiments are performed with the LB films to reveal more details of the crystallization process. LB films are transferred at a surface pressure *π* of 15 mN/m. At this surface pressure, the substrate surface is mostly covered with domains or film without breaking or multilayer formation as confirmed by AFM. Figure 7 shows the GI-WAXS pattern of the LB film of Ethoxy-PPE. A single Bragg reflection in the horizontal direction (q_xy_ axis) and Kiessig fringes [66] in the vertical direction (q_z_ axis) are observed. The horizontal reflection at q_xy_ positions of ~15.1 nm^−1^ (d = 0.41 nm) is related to lateral chain packing of the methylene sequence with a nearest neighbor spacing [41,42] which occurs also as a rotator phase R_II_ known from n-alkanes [67,68,69]. Note that Langmuir films of long-chain hydrocarbon or phospholipids under GI-WAXS investigation show a similar type of Bragg reflection at the horizontal direction with a q_xy_ value in the range of 14.8 to 15.1 nm^−1^, which is typically assigned to side by side chain packing with a nearly vertical rod-like orientation [70]. The Ethoxy-PPE sample was also studied by wide-angle X-ray scattering (WAXS) in the bulk state (see Figure S4). A single Bragg reflection at 2θ ~ 21.91° (q ~ 15.4 nm^−1^) is observed, indicating almost the same nearest neighbor spacing as observed in GI-WAXS. This suggests that the LB film of Ethoxy-PPE possesses a similar chain packing as its bulk state. Melt grown and solvent cast crystals of Ethoxy-PPE also show a single Bragg reflection and the crystal structure was assigned to pseudo-hexagonal [42,71,72]. GI-WAXS pattern of Methyl-PPE-*co*-decadiene has also a single Bragg reflection at a similar q_xy_ position of ~15.1 nm^−1^ as Ethoxy-PPE (Figure S9 of supporting information). The WAXS pattern of this modified PE in bulk state possesses two Bragg reflections at 2θ of ~21.54° and ~23.91° (see Figure S4), which can be identified as an orthorhombic rotator phase R_I_ of the methylene sequence [67,68,69]. This reflection is not visible in the GI-WAXS image, even measuring the sample for a long time. This is because the footprint of GI-WAXS spot is around 0.2 × 10 mm^2^, resulting in a broad distribution in the detected signals due to the scattering from several locations of the film [73]. It is worth mentioning that the crystal structure of these PEs is different, e.g., rotator phase R_II_ for Ethoxy-PPE and orthorhombic rotator phase R_I_ for Methyl-PPE-*co*-decadiene.

Besides, the vertically oriented reflections in the GI-WAXS image along q_z_ axis are the Kiessig fringes [66], which can be used to calculate the film thickness by d = 2*π*/Δq, where d is the layer thickness and Δq is the fringe width, i.e., the difference between two minima or maxima of the fringes. A film thickness of ~2.7 nm is estimated for Ethoxy-PPE, which is consistent with the film thickness obtained by AFM measurement at the same transfer pressure. However, for Methyl-PPE-*co*-decadiene, a film thickness of (5.2 ± 0.5) nm is observed. This value significantly deviates from the value obtained by AFM measurement (~3.2 nm). The origin of this difference is unknown. It should also be noted that the thickness estimation using X-ray measurements and AFM usually result in some differences [74].

## 4. Conclusions

**Figure 8:**
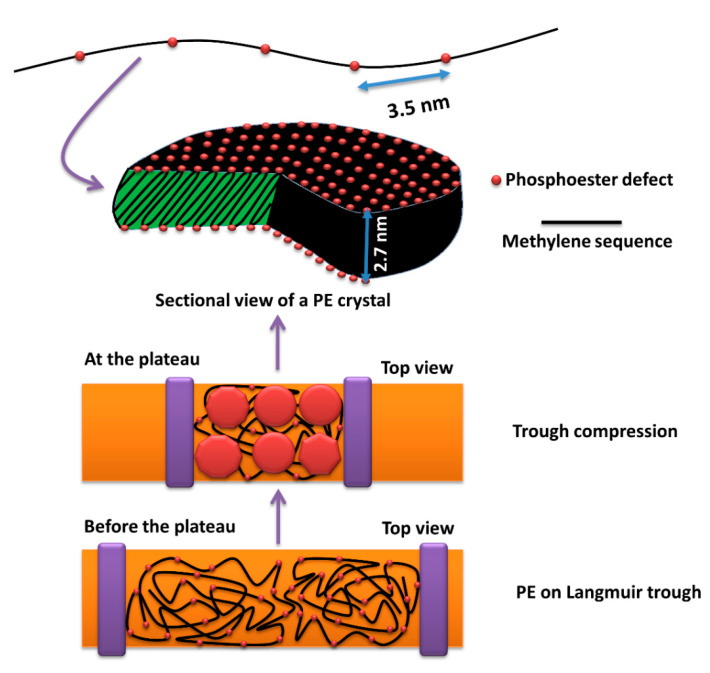
Schematic illustration of the chain organization of Ethoxy-PPE at the air–water interface during compression.

This study shows the possibility to prepare Langmuir/LB film from two different hydrophobic polymers. Using ADMET polymerization, the issue regarding the lack of polar groups in PE chains can be solved. The film morphology can also be tuned when changing the polar groups or increasing the length of methylene sequence between the polar groups (defects). We show the Langmuir/LB film formation of two different PEs (see Table 1), containing regularly spaced polar phosphoester defects in the main chain. The films are prepared by spreading the polymers from chloroform solution on the water surface of a Langmuir trough and compression after solvent evaporation by moving the barriers of the Langmuir trough (Figure 8).

The surface pressure vs. area per repeating unit isotherms are recorded. They show a plateau zone in the surface pressure range of ~(6 to 8) mN/m, assigned to crystalline domain formation by BAM and epifluorescence microscopy. The shape of the domains depends on the defects and the methylene chain length between the defects. PE with ethoxy phosphoester defects shows mostly circular shape domain. The domains turn to a film-like morphology when the chain length between the methyl phosphoester defects increases and when the length of the methylene chains within the PE is different. The thickness of the domains/film is in between ~(2.4 to 3.2) nm, depending on the transfer pressure. Finally, GI-WAXS investigations on the domains or film on solid support show a single Bragg reflection at a q_xy_ position of ~15.1 nm^−1^ known from crystalline PE samples.

## Figures and Tables

**Table 1:** List of the polymers under investigation with their chemical structure, short naming, and number average molar mass *M_n_*.

Polymer Name and Chemical Structure	Abbreviatedas	M_n_* (g/mol)	M_w_/M_n_*
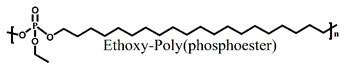	Ethoxy-PPE	9900	2.30
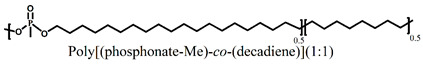	Methyl-PPE-*co*-decadiene	8500	2.08

* Determined by gel permeation chromatography (GPC) (Tetrahydrofuran; THF, Poly(styrene); PS) standard).

## Supplementary Materials

The following are available online at https://www.mdpi.com/2073-4360/12/10/2408/s1, Figure S1: ^1^H NMR spectra of Methyl-PPE-*co*-decadiene, Figure S2: ^31^P NMR spectrum of Methyl-PPE-*co*-decadiene, Figure S3: GPC elugram of Methyl-PPE-*co*-decadiene, Figure S4: DSC and XRD of PEs, Figure S5: FTIR spectra of the investigated PEs and the reactants, Figure S6: BAM image of Methyl-PPE-*co*-decadiene at collapsed phase, Figure S7: *π*-*A* isotherms of Ethoxy-PPE on different subphases, Figure S8: AFM images of the LB film of Methyl-PPE-*co*-decadiene and Figure S9: 2D GI-WAXS pattern of the LB film of Methyl-PPE-*co*-decadiene.
