# Supplementary material for: Crystallization of Poly(ethylene)s with Regular Phosphoester Defects Studied at the Air–Water Interface
Source: Polymers (Basel). 2020 Oct 19;12(10):2408. doi: 10.3390/polym12102408 (PMC7650800; doi:10.3390/polym12102408)
Supplement: Supplementary file 1 [file polymers-12-02408-s001.zip › polymers-966033-supplementary.docx]

**Supporting Information**

# Crystallization of poly(ethylene)s with regular phosphoester defects studied at the air-water interface

Nazmul Hasan^1^, Karsten Busse^1^, Tobias Haider^2^, Frederik R.Wurm^3^ and Jörg Kressler^1*^

^1^Institute of Chemistry, Martin Luther University Halle-Wittenberg, D-06099 Halle, Germany

^2^Max Planck Institute for Polymer Research, Ackermannweg 10, D-55128 Mainz, Germany

^3^Sustainable Polymer Chemistry Group, MESA+ Institute for Nanotechnology, Faculty of Science and Technology, Universiteit Twente, PO Box 217, 7500 AE Enschede, The Netherlands.

*Corresponding author.

E-mail address:[joerg.kressler@chemie.uni-halle.de](mailto:joerg.kressler@chemie.uni-halle.de) (Jörg Kressler)

**Synthesis of Methyl-PPE-*co*-decadiene random copolymer:**

Di(undec-10-en-1-yl) methylphosphonate (500 mg, 1.25 mmol) [1] and 1,9‑decadiene (173 mg, 1.25 mmol) were placed in a Schlenk tube and degassed by three freeze-pump-thaw cycles. Grubbs first generation catalyst (21 mg, 25 µmol) was added under an argon atmosphere with stirring. The catalyst dissolved quickly, yielding a purple liquid. This mixture was heated to 60 °C and a dynamic vacuum was applied until an increase in viscosity was observed. Then full vacuum (4 x 10^−2^ mbar) was applied to further remove the evolving ethylene. After 17 hours another 10 mg of Grubbs first generation catalyst was added and the polymerization proceeded for an additional 24 hours. After that the reaction mixture was cooled down and 5 mL dichloromethane was added to dissolve the polymer. Around 50 mg tris(hydroxymethyl)phosphine and ca. 40 µL triethylamine were added to convert the catalyst to a water-soluble species. After stirring for 3 hours, the solution was further diluted with dichloromethane and then washed with 1x a 2M HCl solution and 3x with H_2_O. The organic phase was then dried over MgSO_4_, filtrated and dichloromethane was removed at a reduced pressure to yield a slightly yellow viscous oil.

Hydrogenation: polymer with a weight of 380 mg was dissolved in 10 mL toluene and charged in a 250 mL ROTH autoclave together with 50 mg of 20 wt% Pd(OH)_2_/C. The solution was then bubbled through argon for 15 minutes. Hydrogenation was performed at room temperature under a hydrogen pressure of 50 bar. The reaction was monitored by ^1^H NMR and stopped in 7 days after completion. Finally, the polymer was obtained as a white powder after the removal of catalyst and solvent.

[1] Steinbach T.; Alexandrino E. M.; Wahlen C.; Landfester K.; Wurm F. R.; Macromolecules 2014, 47, 4884-4893.


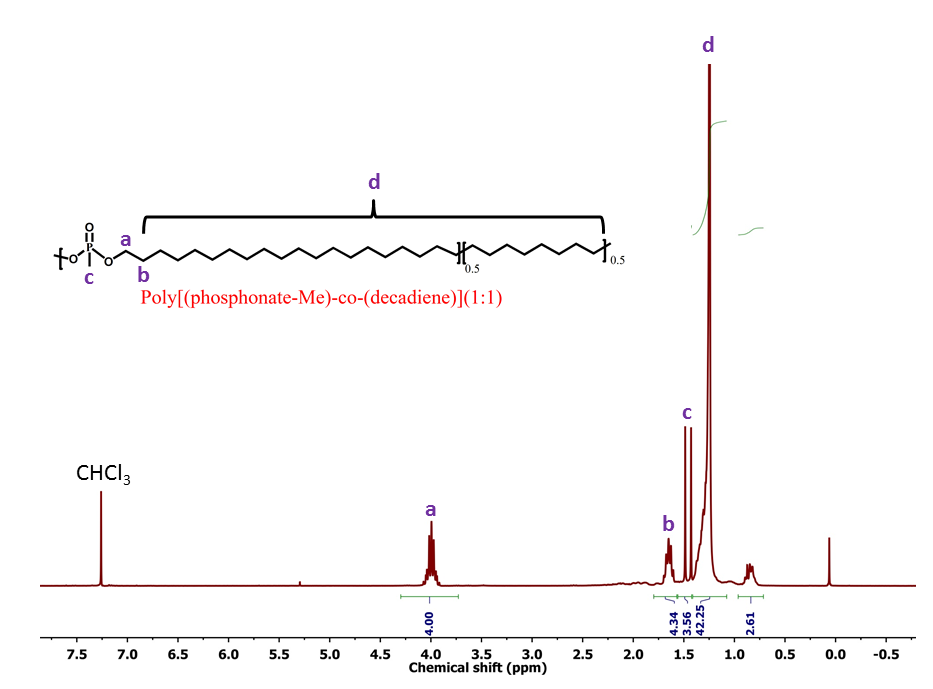


**Figure S1** ^1^H NMR (300 MHz) spectrum of Methyl-PPE-*co*-decadiene in deuterated chloroform with the identified peaks at δ (ppm): 4.17 – 3.83 (m, 4H, P−O−CH_2_), 1.77 – 1.55 (m, 4H, O−CH_2_− CH_2_−), 1.46 (d, J = 17.5 Hz, 3H, P−CH_3_), 1.25 (br s, 48H).


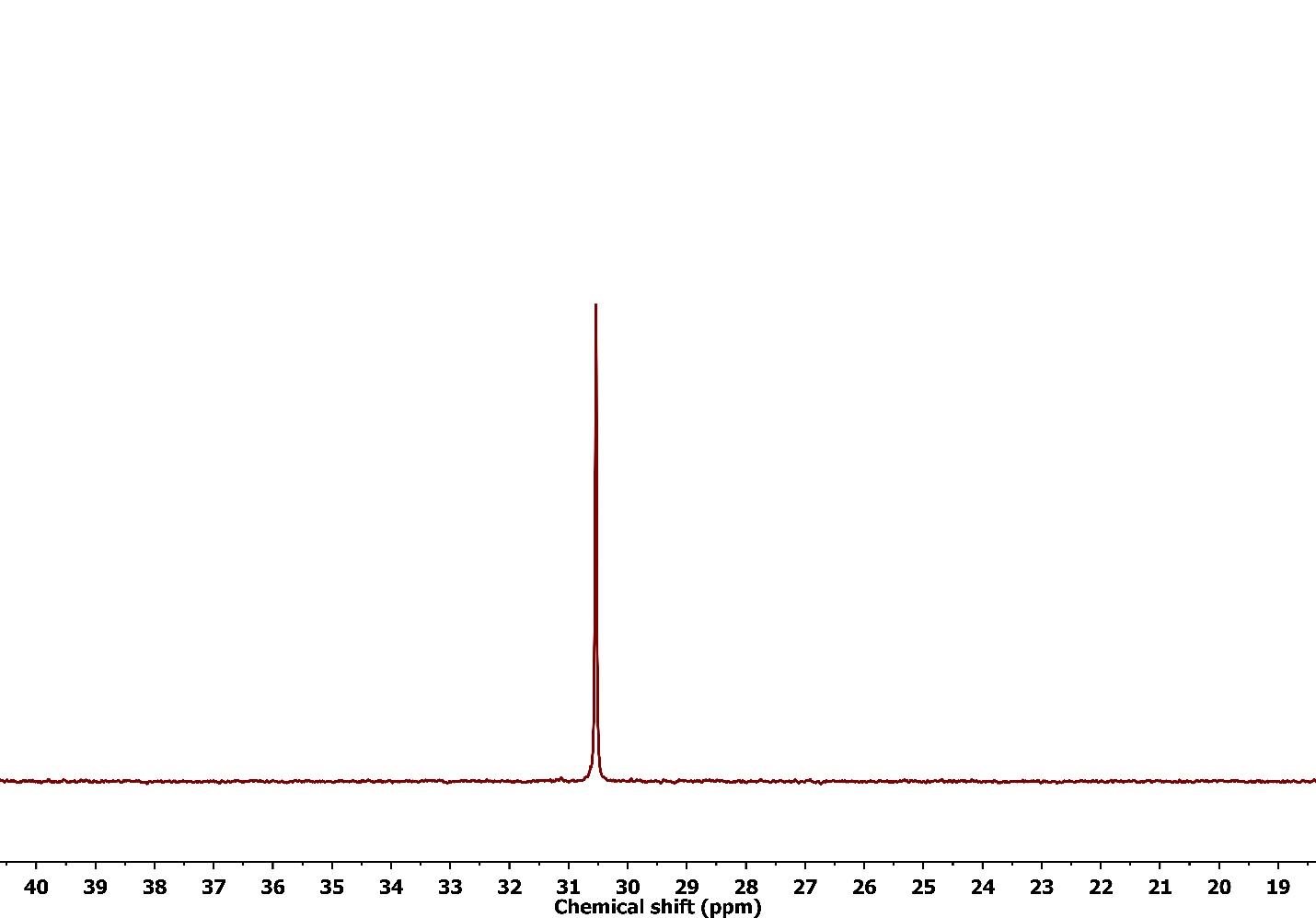


**Figure S2** ^31^P NMR (121 MHz) spectrum of Methyl-PPE-*co*-decadiene in deuterated chloroform.

**Gel permeation chromatography (GPC)**





**Figure S3** GPC elugram of Methyl-PPE-*co*-decadiene in THF.

**DSC and XRD of PEs**

DSC measurements were performed using a METTLER TOLEDO DSC 823 module under continuous nitrogen flow (30 ml/min). Three heating/cooling cycles were used in the range between −100 to180 °C with a heating and cooling rate of 10 °C/min. The DSC data shown here were taken from the second heating curve.

The wide-angle X-ray diffraction patterns were collected with a PANalytical Empyrean Diffractometer in Bragg-Brentano geometry. The instrument was equipped with a position-sensitive PIXcel-3D detector. The samples were placed on zero background silicon substrates in a TTK 450 sample stage from Anton Paar. The measurements were performed at room temperature with Cu Kα radiation (λ = 1.54 Å) in a scan range of 4° < 2θ < 40° with a step size of Δ2θ = 0.013° and a counting time per step of 99 s.


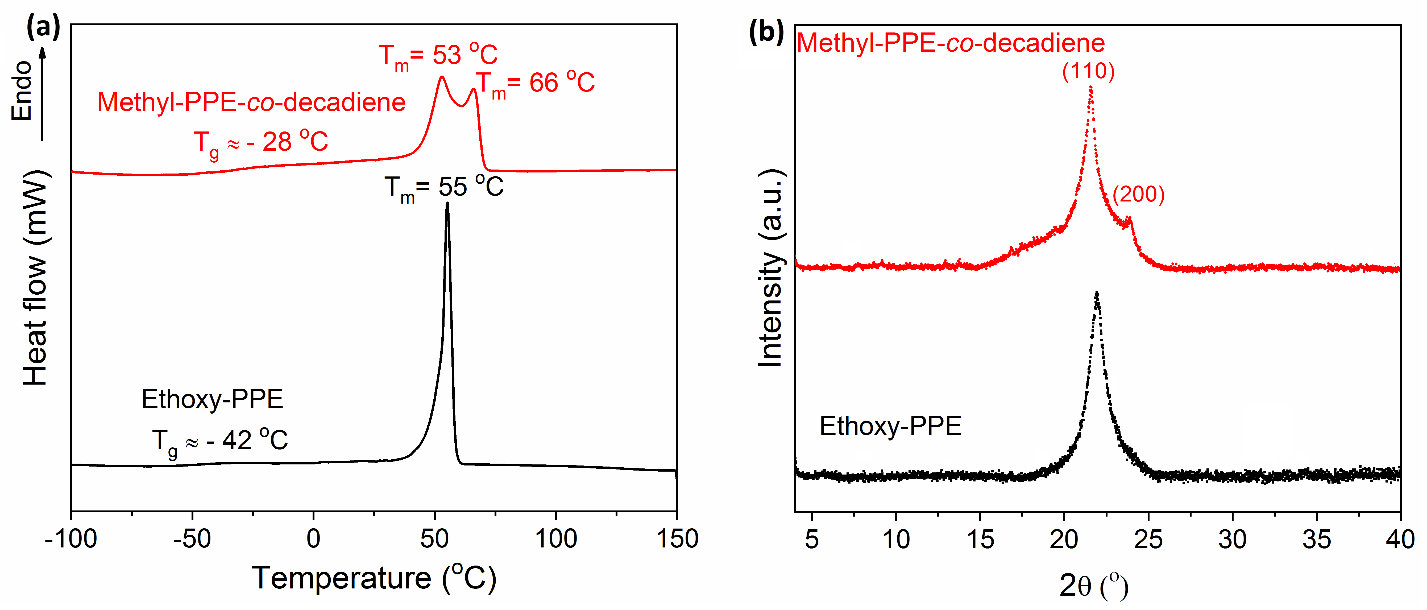


**Figure S4** (a) DSC traces of PEs recorded with 10 °C/min heating rate and (b) WAXS patterns of the same polymers in bulk at room temperature.

**Fourier transform infrared (FTIR) spectroscopy**

A BRUKER Vector 22 (Bruker Optik GmbH, Germany) spectrometer was used to perform FTIR experiments for both PEs and reactants. The experiments were carried out in transmission mode in KBr at room temperature. The spectra were recorded in the wavenumber range of 4000 cm^−1^ to 400 cm^−1^ with 128 scans with a resolution of 4 cm^−1^.

**
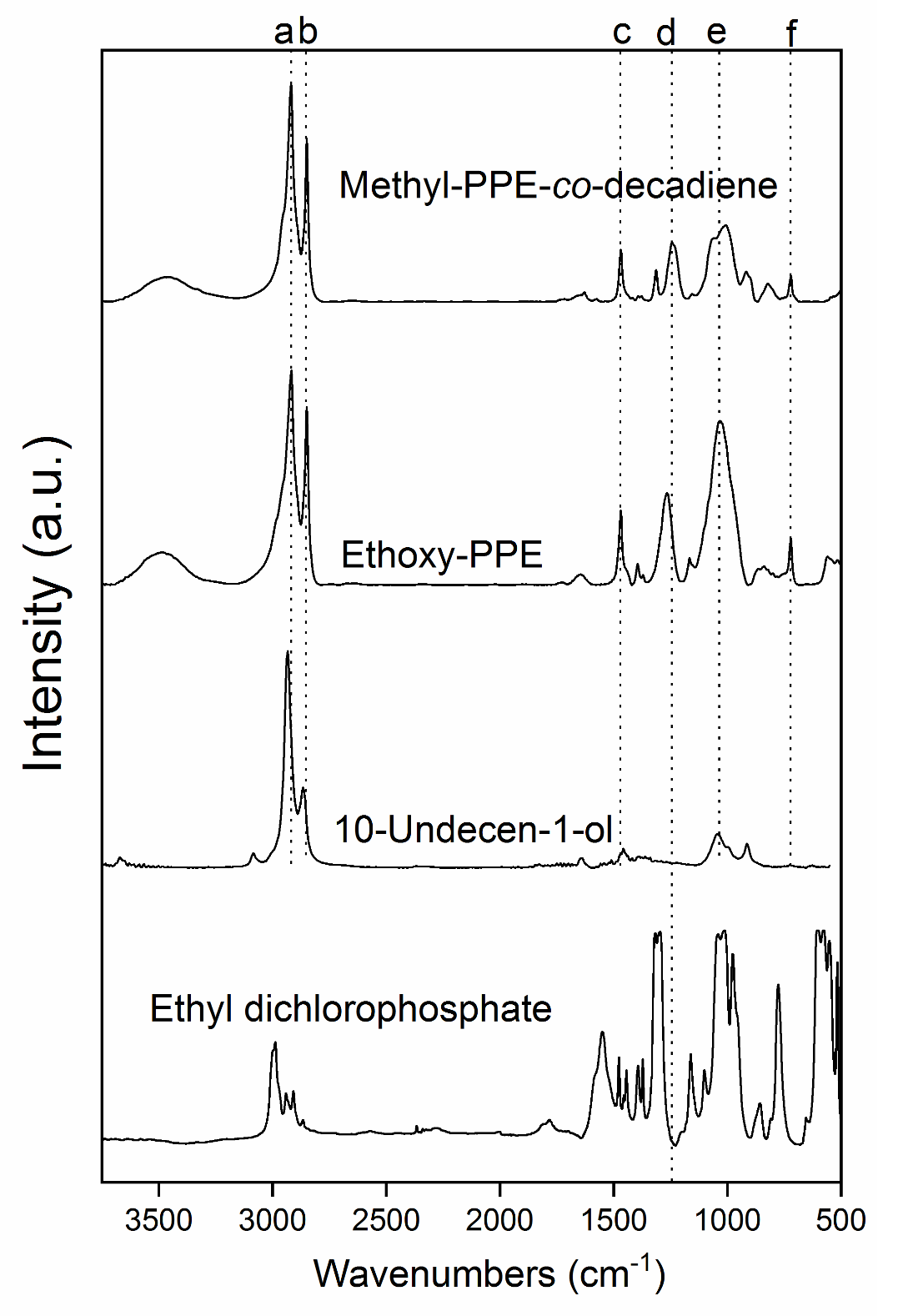
**

**Figure S5** FTIR spectra of the investigated PEs and the reactants acquired in transmission mode in KBr. The data for the reactants are obtained from the online database of the National Institute of Standards and Technology (NIST), USA [2, 3]. The assignment of the bands is as follows: (a) ν_as_(CH_2_), (b) ν_s_(CH_2_), (c) δ_s_(CH_2_), (d) ν(PO), (e) ν(CO), and (f) ρ(CH_2_) [4].

[2] NIST Chemistry WebBook, https://webbook.nist.gov/cgi/cbook.cgi?ID=C1498517&Units=SI&Mask=80#IR-Spec (accessed on 12-10-2020).

[3] NIST Chemistry WebBook, https://webbook.nist.gov/cgi/cbook.cgi?ID=C112436&Units=SI&Mask=80#IR-Spec (accessed on 12-10-2020).

[4] Hasan, N.; Schwieger, C.; Tee, H.T.; Wurm, F.R.; Busse, K.; Kressler, J. Crystallization of a polyphosphoester at the air-water interface. Eur. Polym. J. 2018, 101, 350–357.

**Brewster angle microscope (BAM)**


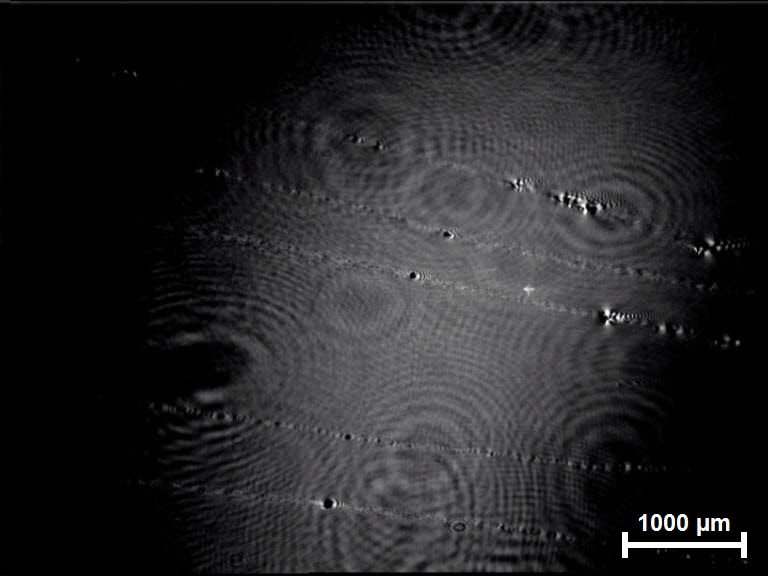


**Figure S6** BAM image of Methyl-PPE-*co*-decadiene captured at a *π* of 33 mN/m (*A* ~ 18 Å^2^). The strips are the collapsed film of this polymer.

**Surface pressure vs area per repeating unit isotherms**

| 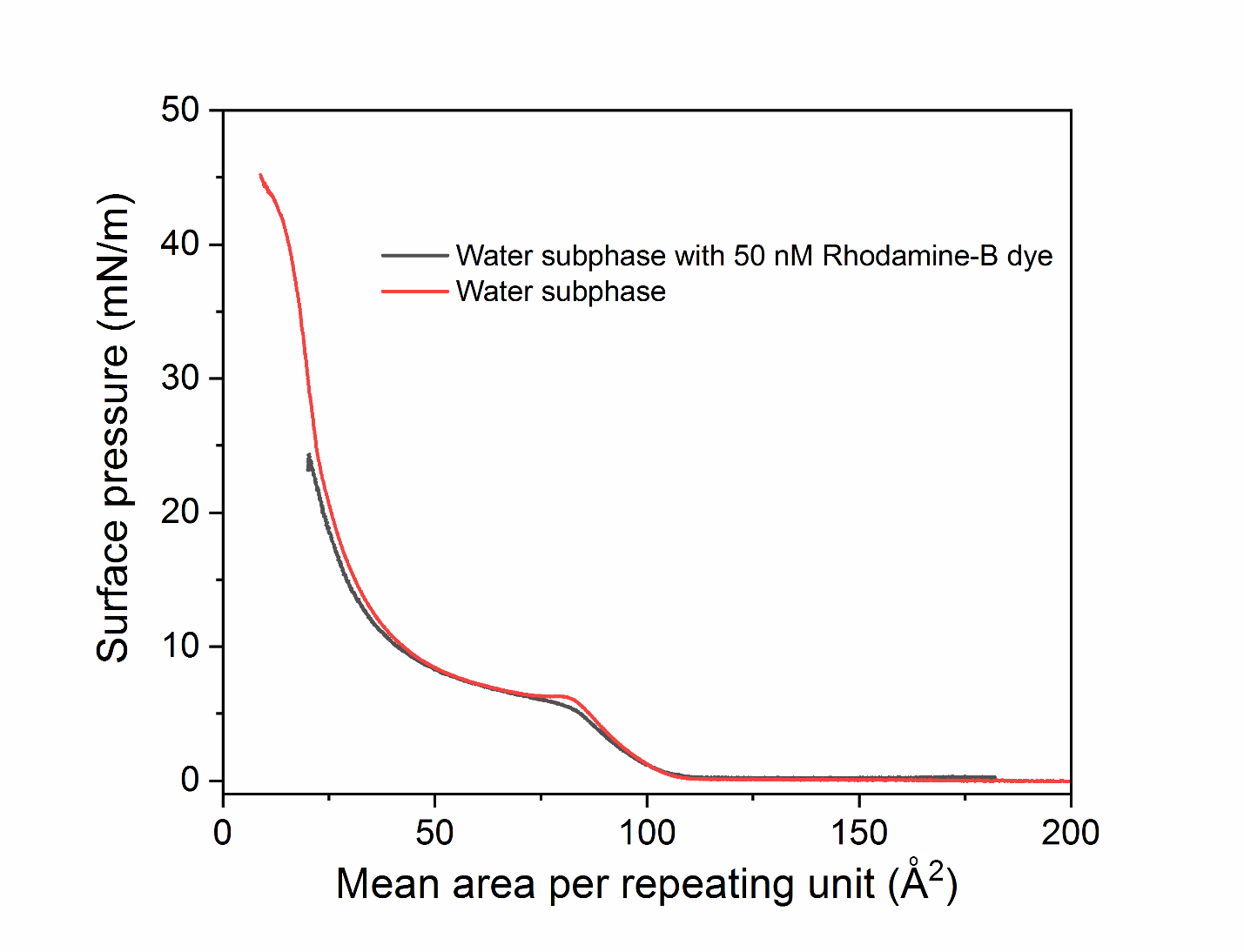 |
| --- |
| **Figure S7** *π-A* isotherms of Ethoxy-PPE on different subphases recorded at a subphase temperature of 20 °C with a compression rate of 50 Å^2^/ (molecule ⋅ min). |

**AFM images of the LB film of Methyl-PPE-*co*-decadiene**

**
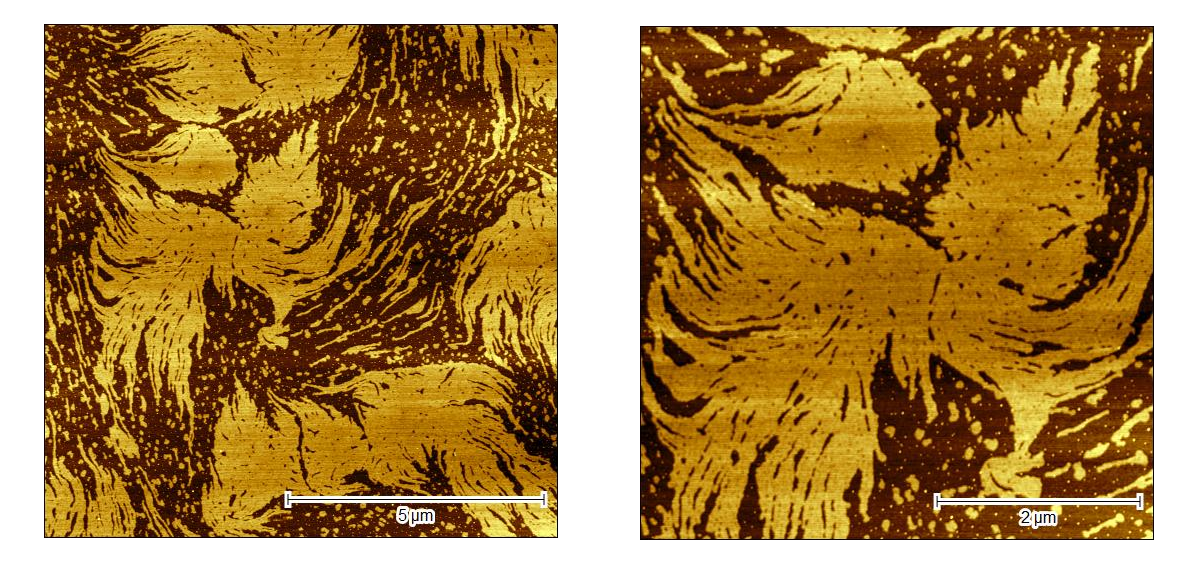
**

**Figure S8** AFM height images of the LB film of Methyl-PPE-*co*-decadiene transferred at a *π* of 10 mN/m.

**Grazing incidence wide angle X-ray scattering**


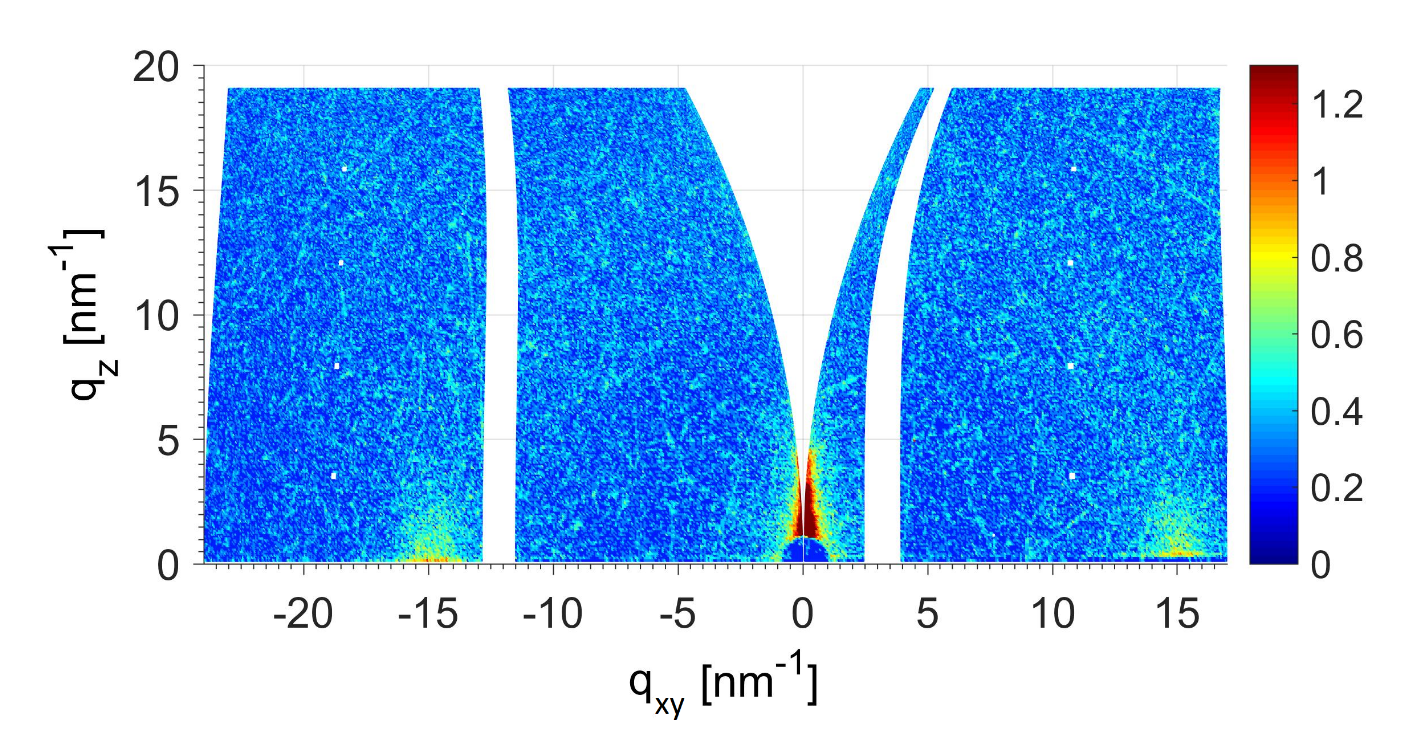


**Figure S9** 2D GI-WAXS pattern of the LB film of Methyl-PPE-*co*-decadiene transferred at a *π* of 15 mN/m. The two vertical bands in the image are the empty space between the detectors and appear as blank.
